# Reflux finding score is associated with gastroesophageal flap valve status in patients with laryngopharyngeal reflux disease: a retrospective study

**DOI:** 10.1038/s41598-019-52349-5

**Published:** 2019-10-31

**Authors:** Wei Wu, Lianyong Li, Changmin Qu, Min Wang, Shuwen Liang, Xiaopei Gao, Xinwei Bao, Lei Wang, Hongdan Liu, Haolun Han, Bingxin Xu, Ying Zhou, Baowei Li, Yiyan Zhang, Gang Wang, Changqing Zhong

**Affiliations:** 1 Department of Otorhinolaryngology Head and Neck Surgery, Chinese People Liberation Army 306th Hospital, Beijing, China; 2 State Environmental Protection Key Laboratory of Environmental Sense Organ Stress and Health, Beijing, China; 3 Department of Gastroenterology, Chinese People Liberation Army 306th Hospital, Beijing, China

**Keywords:** Gastro-oesophageal reflux disease, Oesophagogastroscopy

## Abstract

Endoscopic grading of gastroesophageal flap valve (GEFV) is simple and reproducible and offers useful information for reflux activity. To investigate the potential correlation between GEFV grading and reflux finding score (RFS) in patients with laryngopharyngeal reflux disease (LPRD), 225 consecutive Patients with suspected LPRD who underwent both routine upper gastrointestinal endoscopy and laryngoscope were enrolled in our study. Patients with a RFS of more than 7 were diagnosed with LPRD. The GEFV was graded as I through IV according to Hill’s classification and was classified into two groups: normal GEFV group (grades I and II) and the abnormal GEFV group (grades III and IV). The percent of GEFV grades I to IV was 39.1%, 39.1%, 12.4%, and 9.3%, respectively. Age was significantly related to an abnormal GEFV (p = 0.002). Gender, BMI, smoke and alcohol were not related to GEFV grade. Fifty-one patients (22.67%) had positive RFS. Reflux finding scores were higher in GEFV grades III and IV than I and II (p < 0.05). Endoscopic grading of GEFV is well correlated with reflux finding score in patients with LPRD. This is a simple and useful technique that provides valuable diagnostic information of LPRD.

## Introduction

Gastroesophageal reflux disease GERD is one of the most common GI diseases around the world, with evidence of increasing prevalence in many regions^1^. The impairment of the normal antireflux mechanisms is a main cause for GERD. Furthermore, the gastroesophageal flap valve (GEFV) is a dynamic structure that influences gastroesophageal reflux disease (GERD)^2–6^. Endoscopic grading of the GEFV as proposed by Hill *et al*. is easy and provides useful information about the status of gastroesophageal and gastropharyngeal reflux^2^.

Laryngopharyngeal reflux disease (LPRD), which was first proposed by Koufman *et al*.^7^, is regarded as different from GERD, because LPRD patients do not necessarily have specific symptoms of GERD such as regurgitation or heartburn. Even though the relationship between GERD and the otolaryngological manifestations is still controversial, the two main theories of laryngopharyngeal reflux (LPR) are characterized by acid reflux. The first theory suggests that the fragile mucosa of the larynx and pharynx, in contrast to the oesophagus, is far more susceptible to injury from acid and activated pepsin^8^. The second theory posits that acid stimulates vagally mediated reflexes in the oesophagus, leading to the symptoms of LPR, such as chronic cough and throat clearing sensation^8,9^. Tokashiki *et al*. reported that the LPRD patients showed significantly longer acid reflux time in the upper oesophagus and patients who had LPRD with reflux oesophagitis (RE) experienced more frequent acid exposure in the upper oesophagus than the LPRD without RE^10^.Therefore, there is reason to believe that structural factors, such as GEFV, may affect the pathogenesis of LPRD.

Two instruments, the reflux symptom index (RSI) and reflux finding score (RFS), are commonly used as assessment tools in diagnosing and treating LPR. A study by Kaplan showed that there was a close association between GEFV and reflux symptoms in patients with laryngopharyngeal reflux disease (LPRD)^11^. RFS based on the endolaryngeal inflammatory findings is a scoring system that reduces the subjectivity of the evaluations of LPRD^12^. However, the correlation between RFS and endoscopic assessment of the GEFV has not been investigated. Therefore, in this study, we examine the relationship between GEFV grading and RFS in patients with LPRD.

## Results

### Demographic characteristics of the patients

Total of 225 cases were enrolled into this study, 141 were male, and 84 were female. The median age was 50.63 ± 16.56. Agreement between the two doctors in evaluating the GEFV and RFS is indeed reasonably good with the agreement percentage of 89.33% in GEFV and 88.0% in RFS. Of all the 225 patients, 88 presented with GEFV grade I, 88 with GEFV grade II, 28 with GEFV grade III and 21 with GEFV grade IV. GEFV had no significant correlation with gender, body mass index, smoking and alcohol consumption (p > 0.05). The median ages of patients with normal and abnormal GEFV were 49.52 ± 16.06 and 54.63 ± 17.81, respectively (p = 0.002). Patient characteristics are shown in Table 1.

**Table 1:** Demographic data and gastroesophageal flap valve grade of study subjects.

		Gastroesophageal flap valve grade							P value
		I	II	III	IV	I + II	III + IV	Total	
Gender	Male	57	53	15	16	110	31	141	0.92
	Female	31	35	13	5	66	18	84	
Age		45.60 ± 14.44	53.43 ± 16.73	52.43 ± 15.94	57.57 ± 20.08	49.52 ± 16.06	54.63 ± 17.81	50.63 ± 16.56	0.002
BMI		24.90 ± 3.28	24.53 ± 3.50	23.57 ± 3.87	25.00 ± 3.51	24.71 ± 3.39	24.19 ± 3.75	24.60 ± 3.47	0.33
Smoke	Never	64	75	23	15	139	38	177	0.32
	Occasionally	3	1	1	0	4	1	5	
	Every day	21	12	4	6	33	10	43	
Alcohol	Never	53	60	22	14	113	36	149	0.16
	Occasionally	25	23	6	6	48	12	60	
	Every day	10	5	0	1	15	1	16	

### GEFV and RFS

Of the 174 patients with negative RFS, 32 (21.77%) patients had abnormal GEFV whereas in 51 positive RFS patients, 17 (33.33%) had abnormal GEFV. There was a correlation between GEFV grades and RFS (p = 0.023) (see Table 2).

**Table 2:** Correlation of the prevalence of reflux finding score with gastroesophageal flap valve grade.

RFS	Gastroesophageal flap valve grade						Total
	I	II	III	IV	I + II	III + IV	
≤7	66	76	25	7	142	32	174
>7	22	12	3	14	34	17	51

Spearman correlation p < 0.05 (0.023).

### GEFV and EE

The distribution of GEFV grades and endoscopic findings in oesophagus are shown in Table 3. There were 20 (18.34%), 10 (18.87%) and 19 (30.16%) patients with abnormal GEFV grades in normal, NERD and EE group by endoscopic findings. Abnormal GEFV was significantly more common in patients with oesophagitis compared with those without (p = 0.012).

**Table 3:** Correlation of gastroesophageal flap valve grade with the endoscopic findings in oesophagus.

Endoscopic findings in oesophagus	Gastroesophageal flap valve grade						Total
	I	II	III	IV	I + II	III + IV	
Normal	48	41	15	5	89	20	109
NERD	24	19	6	4	43	10	53
EE	16	28	7	12	44	19	63

Spearman correlation p < 0.05 (0.012).

### RFS and EE

For patients with EE, 46 (35.06%) a negative RFS and 17 (33.33%) had a positive RFS. There was no significant difference on RFS within the groups by endoscopic findings in oesophagus (p > 0.05) (see Table 4).

**Table 4:** Correlation of the prevalence of reflux finding score with the endoscopic findings in oesophagus.

RFS	Endoscopic findings in oesophagus				Total
	Normal	NERD	Normal + NERD	EE	
≤7	84	44	128	46	174
>7	25	9	34	17	51

Spearman correlation p > 0.05 (0.72).

## Discussion

Gastroesophageal reflux disease (GERD), a chronic disorder with increasing prevalence globally, is caused mainly by incompetence of the antireflux barriers at the oesophagogastric junction. The lower oesophageal sphincter (LES) along with the flap valve works together and forms a powerful antireflux barrier. The flap valve is formed by the oblique angle at which the oesophagus enters and integrates with the stomach. Once a system was created to describe and classify GEFV, there has been ongoing research assessing the relationship of endoscopic oesophagitis and gastroesophageal flap valve in patients with symptomatic gastroesophageal reflux^13–15^. In our study, we found that grade of GEFV was correlated with age of the patient and the oesophagitis. The findings that abnormal GEFV (grades III and IV) was more frequent in patients with oesophagitis and elderly patients are consistent with results of previous reports^13–16^. LES pressure was significantly lower and gastroesophageal reflux in the probe were significantly higher in the abnormal GEFV group compared to the normal GEFV group^14^.

Laryngopharyngeal reflux is an extraoesophageal variant of gastroesophageal reflux disease (GERD) that affects the larynx, pharynx, and upper aerodigestive tract. Patients presenting with extraoesophageal reflux–related signs and symptoms are estimated to account for 10% of an otolaryngologist’s practice. A wide spectrum of disorders has been associated with the presence of LPR, including chronic laryngitis, hoarseness, laryngeal carcinoma, globus sensation, cough, subglottic stenosis, vocal process granuloma, and possibly chronic sinusitis. However, at present, there is no validated instrument whose purpose is to document the physical findings and severity of LPR. The available diagnostic methods for LPR include 24 h ambulatory pH monitoring, gastroesophageal endoscopy, laryngoscope, and RSI. The 24 h ambulatory pH monitoring has good sensitivity and specificity, but the clinical application has been limited due its discomfort and its high cost. RSI is a noninvasive method for LPR, however its subjective nature causes the high possibility of response bias in patient self-assessment questionnaires. Laryngoscopy is still the standard for LPR diagnosis accompanied by the RFS. In addition, RFS may accurately document treatment efficacy in patients with LPR^17^. In our present study, we found that increased GEFV grade was significantly associated with an increased reflux finding score, and the frequency of LPR was significantly higher in the abnormal GEFV group compared to the normal GEFV group. Another study by Kaplan also showed that endoscopic grading of GEFV is a simple and useful technique that may provide an accurate diagnosis of laryngopharyngeal and gastroesophageal reflux^11^.

Although LPR is widely regarded as an extraoesophageal manifest of GERD, some researchers did not find significant relationship between LPR and endoscopic oesophagitis and stated that LPR and GERD are not the same diseases^18^. Interestingly, we also found that RFS had no correlation with endoscopic findings in oesophagus even though GEFV was correlated with both RFS and the oesophagitis. In the above-mentioned study^11^, Kaplan did not find any correlation between reflux symptom index and degree of oesophageal mucosal injury according to LA classification. Speculatively, abnormal GEFV plays an important role in GERD and LRP. Ultimately, our findings suggest that LRP may have a more complicated pathogenesis oesophageal motility patterns, the function of pharynx and upper oesophageal sphincter, and mechanisms of airway protection.

To our knowledge, the present study is the first study that evaluates the correlation between GEFV findings and RFS. We displayed the statistically significant relationship between the two most commonly used objective methods, i.e., gastroscopy and laryngoscopy. In recent years, minimally invasive endoscopic intervention of GEFV, such as anti-reflux mucosectomy (ARMS), has been introduced refractory GERD and achieved satisfactory results. Undoubtedly, our study provides theoretical basis for the feasibility of endoscopic treatment of LPR. However, this study has some limitations. Firstly, an obvious limitation of such an analysis is the retrospective, single-centered and non-randomized design inevitably leading to a selection bias. Secondly, oesophageal mucosal injury was not classified according to LA classification due to the small study population. Thirdly, RFS is non-specific. Although we have made some restrictions on the inclusion criteria of cases, it may be positive in some diseases that are difficult to be identified from LPRD, such as allergic rhinitis, vasomotor rhinitis or chronic rhinosinusitis. This could affect the analysis results to some extent. In addition, the patients did not undergo 24 h pH monitoring and RSI evaluation. Therefore, more comprehensive analysis is not possible due to the lack of such data.

In conclusion, the frequency of both GERD and LPRD was significantly higher in the abnormal GEFV group than in the normal GEFV group. We supposed that gastroesophageal endoscopy can be performed to ensure the diagnosis of LPR by evaluating the GEFV. Further research is needed in a larger sample and well-controlled studies are needed to confirm the reliability of this study.

## Methods

### Study design

From September 2017 through September 2018, the data of RFS and GEFV of consecutive patients with suspected LPRD from our hospital were collected and analysed retrospectively in the study. Regardless of whether having reflux and/or heartburn, the patients were enrolled in the study if they presented with at least one of the following symptoms: hoarseness or problem with their voice, throat clearing, excess throat mucus, postnasal drip, chronic cough, breathing difficulties or choking episodes, dysphagia, or discomfort in throat lasting more than a month. All patients underwent both routine upper gastrointestinal endoscopy and TV fibrolaryngoscope after written informed consent was obtained. We excluded patients who had known oesophageal disease such as cancer, achalasia, stricture, active peptic ulcer disease or prior history of upper gastrointestinal surgery; used antibiotics or proton pump inhibitors (PPIs), mucosal protective agents or gastroprokinetic agents within 1 week; or had difficulty tolerating upper gastrointestinal endoscopy and TV fibrolaryngoscope. This study was approved by the Ethics Committee of the 306^th^ Hospital (Approved Document Number: K2017–06), and all the patients provided written informed consent for the endoscopy and TV fibrolaryngoscope and use of data for research purposes. And all experiments were performed in accordance with relevant guidelines and regulations.

### Endoscopy and GEFV

**Figure 1:**
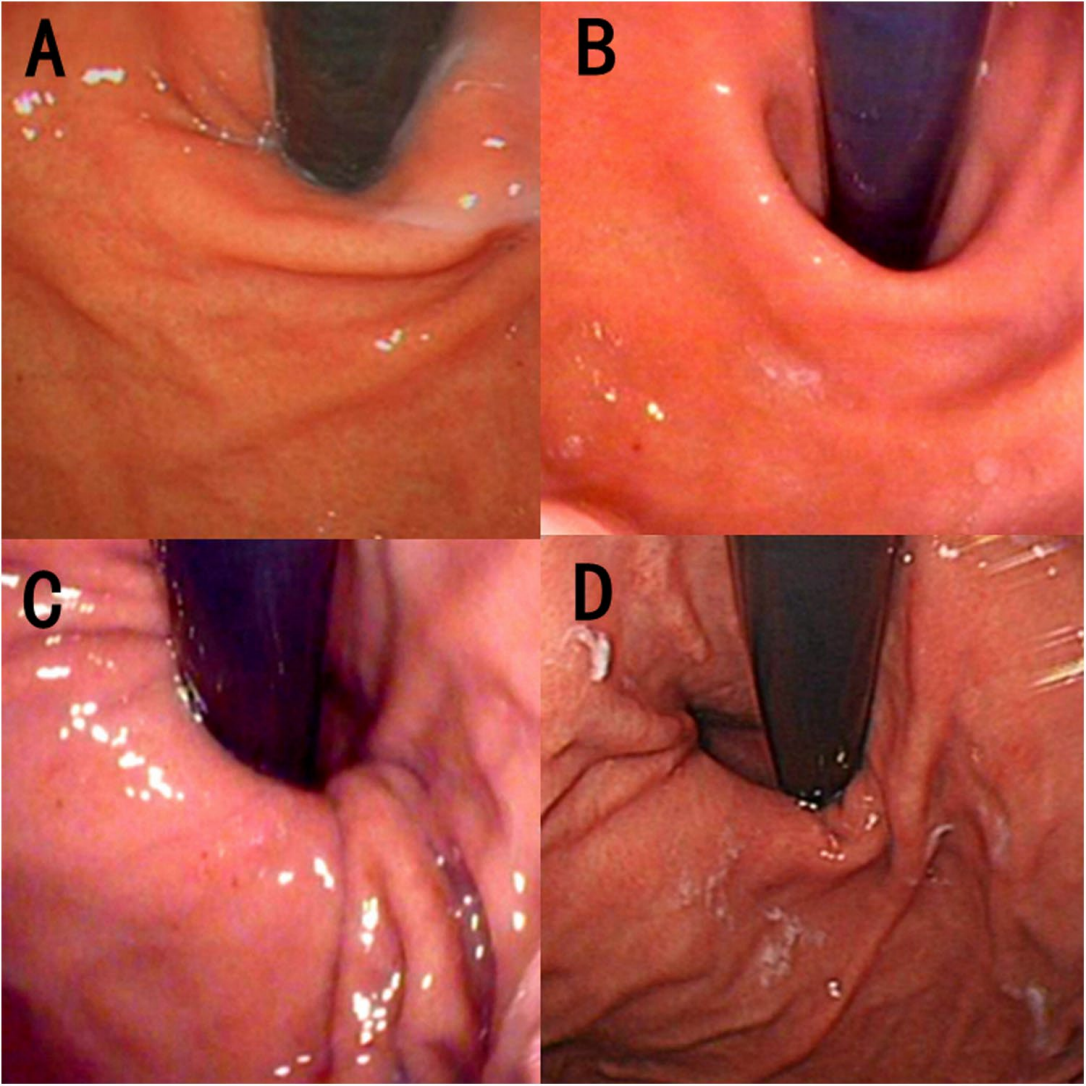
GEFV by Hill’s Classification. (**A**) Grade I: a prominent fold of tissue along the lesser curvature and closely apposed to the endoscope; (**B**) Grade II: the fold is present, but there are periods of opening and rapid closing around the scope. (**C**) Grade III: the ridge is barely present, and there is often failure to close around the scope. (**D**) Grade IV, the muscular ridge is absent, and the gastroesophageal area continuously remains open. A hiatal hernia is always present.

Upper gastrointestinal endoscopy was performed using flexible video endoscopy XQ260/H260 (Olympus Co. Ltd, Tokyo, Japan) or EG29-i10 (Pentax Medical, Tokyo, Japan) under intravenous anaesthesia in each patient. The GEFV was inspected with a retroflexed endoscope and graded I to IV according to the Hill classification. Examples of Hill flap valve grades I-IV are shown in Fig. 1. All endoscopic procedures were performed by experienced endoscopists, and the GEFVs of all patients in this study were evaluated by two endoscopists (L.Y.L & M.W). Disagreements among the evaluators were resolved by discussion. GEFV grades I and II were regarded as normal, while grades III and IV were abnormal. Erosive oesophagitis (EE) was defined as presence of oesophageal mucosal breaks. Nonerosive reflux disease (NERD) was defined as the presence of classic GERD symptoms in the absence of oesophageal mucosal injury during upper endoscopy.

### Reflux finding score

RFS rating scales were developed by Belafsky^17^ for the assessment of the patients with LPR, as shown in Table 5. The reflux finding score is an 8-item clinical severity scale used to interpret the most common laryngoscopic findings related to LPR. RFS was analyzed and evaluated by 2 senior doctors (W.W & G.W). The final figure of RFS is an average of these two sets of data. Patients with a RFS of higher than 7 were diagnosed with LPRD.

**Table 5:** Reflux finding score rating scales.

Finding	Score
Subglottic oedema	0 = absent
	2 = present
Ventricular obliteration	2 = partial
	4 = complete
Erythema/hyperaemia	2 = arytenoids only
	4 = diffuse
Vocal cord oedema	1 = mild
	2 = moderate
	3 = severe
	4 = polypoid
Diffuse laryngeal oedema	1 = mild
	2 = moderate
	3 = severe
	4 = obstructing
Posterior commissure hypertrophy	1 = mild
	2 = moderate
	3 = severe
	4 = obstructing
Granuloma/granulation	0 = absent
	2 = present
Thick endolaryngeal mucus/other	0 = absent
	2 = present

### Statistical analysis

The prevalence rates of RFS and oesophagitis and frequency of abnormal GEFV were calculated. Categorical variables were analysed using Pearson’s chi-squared test, and continuous variables were analysed using Student’s t test or one-way analysis of variance. The Spearman’s rank correlation coefficient was used to measure the strength and direction of association between ordinal variables. A p value < 0.05 was considered statistically significant. Data analysis was generated using Statistical Package for the Social Sciences, version 19.0 (SPSS, Chicago, IL, USA).

## Data Availability

The datasets analyzed during the current study are available from the corresponding author on reasonable request.
